# A pilot randomized controlled trial of a telemedicine psychosocial intervention to improve symptom management in adults with long COVID: the COPE study protocol

**DOI:** 10.1186/s40814-024-01515-2

**Published:** 2024-06-17

**Authors:** Lindsey M. Knowles, Mehr Grewal, Sydney A. Drever, Jeanne M. Hoffman, Janna L. Friedly, Tracy E. Herring

**Affiliations:** 1 Department of Rehabilitation Medicine, University of Washington School of Medicine, Seattle, WA 98104 USA; 2 Worth A Shot Initiative, Bellevue, WA USA

**Keywords:** Long COVID, Self-management, Rehabilitation, Multidisciplinary treatment, Telemedicine, Randomized controlled trial

## Abstract

**Background:**

Long COVID is a serious public health concern due to its high prevalence and potentially debilitating symptoms. Symptoms may include fatigue, dyspnea, cognitive problems, insomnia, anxiety, and depression. There is currently no cure for long COVID, and the average length of recovery and proportion of patients who fully recover are still unknown. Subsequently, there is a critical need to improve function. Research in other chronic conditions suggests that psychosocial self-management interventions reduce symptom severity and interference with functioning. We describe the design of our study to examine the feasibility, acceptability, appropriateness, and preliminary efficacy of an intervention designed to improve symptom management and coping in adults with long COVID.

**Methods:**

This pilot trial (*N* = 50) uses a pragmatic, randomized two-group parallel design set within the University of Washington Post-COVID Rehabilitation and Recovery Clinic. The self-management intervention is a 6-week, group-based telemedicine intervention that teaches evidence-based strategies to manage common symptoms and improve stress management as well as communication and self-advocacy. The comparator is a wait-list control. Participants complete self-report measures of the primary and secondary outcomes at baseline and post-treatment/wait-list. Primary outcomes include intervention feasibility, acceptability, and appropriateness. Secondary outcomes include Patient-Reported Outcomes Measurement Information System measures of fatigue, sleep disturbance, cognitive difficulties, self-efficacy, pain interference, depression and anxiety symptoms, and a measure of long COVID symptoms and impression of change. At post-intervention, intervention participants also complete a qualitative interview to inform intervention refinement. Quantitative data will be examined using descriptive and statistical analysis including *t*-tests and chi-square tests to compare the intervention and wait-list groups on secondary outcomes. Qualitative data will be analyzed using the rigorous and accelerated data reduction technique (RADaR).

**Discussion:**

Results of this pilot randomized controlled trial will characterize the feasibility, acceptability, and appropriateness of the self-management intervention and inform intervention refinement necessary prior to further testing. Long COVID is a public health concern, and rehabilitation approaches that equip patients to manage symptoms may improve patient function and quality of life and reduce burden on the health care system.

**Trial registration:**

NCT05658536. December 16, 2022.

**Supplementary Information:**

The online version contains supplementary material available at 10.1186/s40814-024-01515-2.

## Introduction

The World Health Organization defines long COVID as “the continuation or development of new symptoms three months after the initial SARS-CoV-2 infection, with these symptoms lasting for at least 2 months with no other explanation” [1]. Long COVID poses a serious public health concern as 1 in 13 American adults report experiencing long COVID symptoms [2]. People with long COVID can present with a variety of symptoms ranging from mild to debilitating in severity. Some of the most common and disabling symptoms include fatigue, dyspnea, cognitive problems, pain-related symptoms, and insomnia. In addition, many adults with long COVID report anxiety and depression [3, 4].

Compared to other chronic health conditions, long COVID is new and subsequently less researched. There is currently no known cure for long COVID, and the average length of recovery as well as the proportion of patients who fully recover is still unknown. Given the variability and chronicity of long COVID symptoms, there is a critical need for intervention research to improve symptom management and functioning. Psychosocial self-management interventions could be particularly beneficial for targeting the various biopsychosocial factors that influence long COVID symptoms as well as the distress associated with living with variable symptoms and a relatively novel health condition [5]. Self-management has been defined by Barlow and colleagues [6] as “the individual's ability to manage the symptoms, treatment, physical and psychosocial consequences and life style changes inherent in living with a chronic condition.” Self-management is considered a valuable approach for the management of complex chronic illness and multiple morbidity. For example, self-management is one of four goals in a strategic framework for improving the health status of people with multiple chronic conditions published by the Department of Health and Human Services [7]. Research on patient populations with similar symptom profiles as long COVID (e.g., myalgic encephalomyelitis/chronic fatigue syndrome, multiple sclerosis) suggests that psychosocial interventions focused on self-management can reduce symptom severity and interference with functioning and improve coping [8, 9].

Self-management interventions equip and empower people with chronic health conditions and/or disability to actively manage their symptoms by providing them with strategies that they can use in their day-to-day lives [10, 11]. For example, these strategies may include pacing and energy conservation to reduce or manage fatigue and pain, practicing breathing exercises to improve shortness of breath and related anxiety, applying attention strategies to improve cognition, using cognitive behavioral techniques to change unhelpful patterns of thinking, enlisting social support to improve coping, engaging in goal-directed behavior aligned with values to improve mood, participating in mindfulness and relaxation practices to reduce stress and support mood, and improving sleep hygiene to optimize sleep, to name a few [12]. Furthermore, telemedicine is a viable platform for delivering self-management interventions [13]. Telemedicine is convenient, accessible, and cost-effective, has high patient satisfaction ratings, and reduces the risk for transmission of COVID-19 [14–16].

The mixed-methods randomized controlled trial (RCT) described here was developed in response to the critical need to advance research on effective psychosocial rehabilitation for adults with long COVID. We describe a pragmatic, randomized two-group parallel design to examine the feasibility, acceptability, and appropriateness and explore the preliminary efficacy of a 6-week telemedicine self-management group intervention for adults with long COVID. The self-management intervention will be compared to a wait-list control condition (delayed treatment) because there is currently no standard rehabilitation intervention for adults with long COVID.

Based on the National Institutes of Health (NIH) Stage Model of Behavioral Intervention Development [17], this pilot RCT is consistent with Stage 1 research activities focused on pilot testing and refining the intervention. Specifically, this is the first trial in a program of research aimed at developing and optimizing a psychosocial self-management intervention for adults with long COVID. The intervention is based on evidence-based self-management treatments for other rehabilitation patient populations (e.g., multiple sclerosis) and is already being delivered at the University of Washington (UW) due to high need and demand. Nevertheless, an iterative multistage approach is necessary to test and refine the intervention to be maximally effective and implementable in the intended treatment setting [17]. This pilot RCT is also pragmatic in that it is being conducted and evaluated in the intended treatment setting — an overburdened long COVID clinic in an academic medical center (AMC) setting with AMC providers.

This pilot RCT has three aims designed to inform the overall goal of intervention refinement. The first aim is to examine the feasibility, acceptability, and appropriateness of a telemedicine group self-management intervention designed to improve symptom management and coping in adults with long COVID. We hypothesize that the intervention will demonstrate feasibility, acceptability, and appropriateness, and that average session attendance will be comparable to attendance in RCTs of psychosocial interventions in other rehabilitation populations (43–93%; [18, 19]). The second aim is to explore the preliminary efficacy of the intervention for improving long COVID symptoms. We hypothesize that intervention participants will report greater pre- to posttreatment improvements in long COVID symptoms and psychosocial outcomes as compared to wait-list participants. The third aim is to use qualitative data from interviews with intervention participants to understand their perceptions of the feasibility, acceptability, appropriateness, and perceived efficacy of the intervention to improve the intervention. We do not have a priori hypotheses regarding intervention participant feedback.

## Methods

### Study design overview

This pilot trial uses a pragmatic, randomized two-group parallel design to examine the feasibility, acceptability, appropriateness, and preliminary efficacy of a 6-week telemedicine group self-management intervention for adults with long COVID. The intervention is aimed at improving symptom management and coping in adults with long COVID. Participants are randomized to the intervention group or a wait-list control group. All study methods and procedures have been approved by the UW Institutional Review Board (IRB). The trial is registered on ClinicalTrials.gov with the identifier: NCT05658536. See Table 1 for SPIRIT flow diagram for participant timeline. See supplementary table for SPIRIT Checklist.

**Table 1:** Schedule of enrollment, interventions, and assessments (SPIRIT 2013 guidelines)

	**Study period**		
	**Enrollment**	**Allocation**	**Post-allocation** **6 weeks (post-treatment/wait-list)**
**Timepoint**	***-t***_***1***_	**0**	***t***_***1***_
**Enrollment**			
** Eligibility screen**	X		
** Informed consent**	X		
** Allocation**		X	
**Conditions**			
*** Telemedicine self-management group***		X	
*** Wait-list control***		X	
**Assessments**			
** Primary outcomes**			
Acceptability of Intervention Measure [20]			X*
Intervention Appropriateness Measure [20]			X*
Feasibility of Intervention Measure [20]			X*
** Secondary outcomes**			
Depression Short Form 8b** [21]		X	X
Anxiety Short Form 8a** [21]		X	X
Fatigue Short Form 8a** [21]		X	X
Sleep Disturbance Short Form 8a** [21]		X	X
Cognitive Function — Abilities Short Form 8a** [21]		X	X
Self-Efficacy for Managing Chronic Conditions — Managing Symptoms Short Form 8a** [21]		X	X
Ability to Participate in Social Roles and Activities Short Form 8a** [21]		X	X
Pain Interference Short Form 8a** [21]		X	X
Long COVID symptom and impact tools [22]		X	X
Credibility/expectancy questionnaire [23]		X*	
Patient Global Impression of Change scale [24]			X*

^*^Completed by intervention participants only

**Patient-Reported Outcomes Measurement Information System (PROMIS)

### Study setting

This trial aims to enroll and randomize 50 participants who receive care at the UW Post-COVID Rehabilitation and Recovery Clinic within the state of Washington. This is a high-demand outpatient clinic housed within an AMC that has served over 2500 patients since opening in 2020. Assuming a 10% dropout rate, a total of 50 participants will yield 20 completers in each study condition. This sample size was selected to characterize the intervention’s feasibility, acceptability, appropriateness, and preliminary efficacy to inform intervention improvement. A sample size of 20 participants in each condition (accounting for attrition) is appropriate given the specificity of our qualitative research questions [25, 26]. Likewise, this sample size is appropriate for estimating the proportion of participants who rate the intervention as feasible, acceptable, and appropriate. The lower bound of the 95% CI will be above 60% if at least 17 out of 20 (85%) of intervention participants rate the intervention as feasible, acceptable, or appropriate (Supplemental Fig. 1), which is a conservative estimate given satisfaction ratings for similar rehabilitation interventions [27].

### Participant eligibility and recruitment procedures

For this pragmatic pilot trial, inclusion and exclusion criteria were selected to reflect clinical care in the UW Post-COVID Rehabilitation and Recovery Clinic and to maximize generalizability such that a broad range of patients with long COVID engaged in care in a similar multidisciplinary clinic would be represented. Inclusion criteria are that participants must be at least 18 years of age; reside in the state; have a long COVID diagnosis; be able to read, speak, and understand English; and have reliable telephone and Internet access. Exclusion criteria are severe cognitive impairment as evidenced by two or more errors on the 6-item cognitive screener [28] and current participation in other psychosocial treatment primarily for a long COVID symptom(s) or problem(s).

The UW Post-COVID Rehabilitation and Recovery Clinic serves as the recruitment site. Prospective participants are identified through the wait-list for the existing telemedicine group self-management intervention. Prospective participants are sent an approach letter via email or physical mailing that indicates why they are being approached and how study staff received their name and contact information. Study staff call prospective participants within 1 week after sending the approach letter. Interested patients may also contact research staff directly after receiving the approach letter. Prospective participants may also be identified and referred to the study by providers who are already referring patients to the telemedicine group-based self-management intervention.

### Screening and baseline assessments

The study coordinator provides a brief description of the study to prospective participants over the phone. Prospective participants who are interested in enrolling in the study are then screened for eligibility (see inclusion/exclusion criteria above). Eligible prospective participants complete the informed consent process to enroll into the study. Following enrollment, participants are asked to confirm their availability for upcoming self-management groups which are scheduled based on provider availability. If participants are available for the upcoming group schedule, participants complete a brief reassessment of eligibility, confirming that they have not participated in any other similar self-management programs targeting post-COVID symptoms. Within 2 weeks of the group’s Session 1 date, participants are randomly assigned to the intervention group or the wait-list control group. If availability is not confirmed, the study coordinator contacts the participants to confirm availability for the next group(s), as soon as that schedule is available from the clinic. Following randomization, participants complete a battery of self-report baseline measures. See Table 1 for study schedule and outcome measures. Study data are collected and managed using REDCap electronic data capture tools hosted at the UW [29, 30].

### Self-management intervention

Rehabilitation psychologists at the UW and the VA Puget Sound Health Care System developed a six-session group self-management intervention for patients with long COVID. These groups are currently offered to patients receiving care in the UW Post-COVID Rehabilitation and Recovery Clinic. This intervention teaches participants behavioral strategies known to be helpful in managing symptoms in patient populations with similar symptom profiles as long COVID (e.g., myalgic encephalomyelitis, multiple sclerosis), including fatigue, memory and attention problems, insomnia, dyspnea, coping with anxiety and uncertainty, and stress management [14, 15]. See Table 2 for a description of intervention content by session. The intervention is delivered via telemedicine by licensed rehabilitation psychologists. The rehabilitation psychologists follow the treatment manual and cover the major components of each session, which are highlighted with fidelity statements. Fidelity statements are bolded in the treatment manual and help ensure that the rehabilitation psychologists are covering the essential content. An example of a fidelity statement is “To ensure fidelity say something like the following: ‘In this group, we will apply the biopsychosocial model to many of your symptoms to identify factors that may contribute to the presence of your symptoms as well as to understand and apply strategies to improve your symptoms or how you cope with your symptoms’”). The rehabilitation psychologists meet monthly for case consultation via video conference.

**Table 2:** Major components of the manualized self-management intervention for long COVID

Session	Session content
1	*Introduction to long COVID and the biopsychosocial model* Information and discussion on the following: long COVID and related symptoms, biopsychosocial model and self-management philosophy, and dyspnea Skill: diaphragmatic breathing
2	*Energy management* Information and discussion on the following: long COVID fatigue, cycle of deconditioning, assessing and reestablishing priorities Skill: pacing for energy management
3	*Enhancing sleep, memory, and attention* Information and discussion on the following: sleep problems, memory and attention difficulties Skill: sleep hygiene and strategies to improve attention
4	*Anxiety and coping with uncertainty* Information and discussion on the following: anxiety and emotional responses to COVID-19 and long COVID, discernment for effective coping, mindfulness Skill: coping with controllable and uncontrollable stressors, grounding mindfulness exercise
5	*Stress management* Information and discussion on the following: stress associated with long COVID, cognitive behavioral model, and related strategies to enhance coping with stress Skill: thought challenging, coping thoughts
6	*Enhancing communication* Information and discussion on the following: effective communication with healthcare providers for long COVID care, communication with family and friends about long COVID and symptom management, effective goal setting Skill: communication strategies, goal setting that is specific, measurable, achievable, relevant, time-bound (SMART)

### Randomization and procedures to minimize bias

Participants are randomized using a 1:1 randomization to treatment versus wait-list control condition system. When participants confirm availability for an upcoming clinic group schedule and are confirmed eligible following the re-assessment of Eligibility, participants are 1:1 randomized to either the treatment experimental condition or wait-list control condition. The order of randomization is determined by the order in which participants chronologically completed the re-assessment of eligibility. To minimize bias, the clinicians delivering the intervention are blinded to the research participation status of their group members, as some group members will be enrolled in the study while others will not be. In addition, the REDCap system automatically sends participants baseline and post-treatment/wait-list survey links, where the participant can directly enter their data. As a result, the potential for study staff biases to interfere with data collection is avoided.

### Data collection procedures and measures

Participants complete self-report measures via REDCap at baseline (prior to starting the treatment/wait-list period) and at post-treatment/wait-list after completing the six-session intervention group or 6-week wait period. For data capture and storage, this study uses REDCap, a secure, password-protected, and HIPAA compliant web-based data platform hosted by the UW Institute of Translational Health Sciences [29, 30].

The primary and secondary outcomes are listed in Table 1. Primary outcomes include the Feasibility of Intervention Measure (FIM), Acceptability of Intervention Measure (AIM), and Intervention Appropriateness Measure (IAM) [20]. Each measure consists of four items that are rated on a 5-point ordinal scale assessing the degree to which an intervention is feasible (e.g., “this intervention seems doable”), agreeable (e.g., “I like this intervention”), or appropriate (e.g., “this intervention seems fitting”). A score greater than 3 (“neither agree nor disagree”) will be used as the cutoff for a dichotomous rating of feasibility, acceptability, and appropriateness. The FIM, AIM, and IAM have shown excellent reliability as well as substantive and discriminant content validity [20]. Secondary outcomes include Patient-Reported Outcomes Measurement Information System (PROMIS) measures of fatigue, sleep disturbance, cognitive difficulties, self-efficacy, pain interference, depression and anxiety symptoms [21], and a long COVID symptom and impact tools measure [22] and Patient Global Impression of Change scale [24].

In addition, participants randomized to the self-management intervention (*n* = 25) complete a 45-minute semi-structured interview with research staff within one month after the final session. The interviewer queries participants about the feasibility, acceptability, and appropriateness of the intervention as well as suggestions for improving the intervention across these areas. Participants are also asked about the perceived efficacy of the intervention and how the intervention could be improved to increase efficacy. Last, the interviewer queries participants’ impressions of the intervention workbook and elicit suggestions for improvement. Data is transcribed via Rev, a secure online transcription service [31].

### Data and safety monitoring

The principal investigators provide oversight of the trial in collaboration with study staff to assure adequate protection of the rights of human subjects. They monitor progress of the project, protocol adherence, substantial protocol amendments, and participant safety including adverse events on an ongoing basis in weekly meetings and submit a report to the IRB twice annually. Because this study does not use investigational drugs or devices and poses minimal risk to participants, a data and safety monitoring board, which is required for most United States Food and Drug Administration studies, was not deemed necessary. Instead, the above process serves two purposes: it accommodates the expected low-base rate of problems and allows for the earlier identification of issues more appropriately than a data and safety monitoring board that would meet at an arbitrary regular interval. Likewise, there is no auditing process in place beyond the IRB standard procedures of potentially conducting an audit during the course of the research or after the project is completed. Regarding provisions for ancillary and posttrial care, participants in the self-management group are passively monitored for suicidal ideation. If a group member reports suicidal ideation, the licensed psychologist leading the group provides appropriate follow-up including risk assessment, safety planning, and provision of resources depending on level of risk.

*Note: Future tense will be used from now until the* Discussion*section, as we have yet to conduct the statistical analysis plan*

We will make the data and associated documentation available to users only under a data-sharing agreement that provides for the following: (1) a commitment to using the data only for research purposes and not to identify any individual participant, (2) a commitment to securing the data using appropriate computer technology, and (3) a commitment to destroying or returning the data after analyses are completed.

### Statistical analysis plan

An intent-to-treat (ITT) approach will be used for all quantitative analyses. If participants are missing data at the individual-item level of a multi-item scale, we will impute the missing data using the within-group mean scores for the scale; however, a score will not be imputed if the participant is missing ≥ 40% data in a scale.

First, we will compute descriptive statistics to characterize the sample in terms of demographic and condition-related factors. To assess the first study aim, we will calculate the means and standard deviations of the AIM, IAM, and FIM and quantify the proportion of participants who rate the intervention as feasible, acceptable, and appropriate (average score greater than 3). We will quantify adherence as the percentage of sessions attended out of six sessions. These statistics will be used to contextualize the qualitative results and help inform the extent of modifications necessary to improve the feasibility, acceptability, and appropriateness of the intervention for adults with long COVID.

The second study aim to examine intervention effects is exploratory. We will determine whether randomization was successful by using *t*-tests and chi-square tests to compare the intervention and wait-list groups on a variety of demographic and baseline outcome variables. Any variables that differ between the groups will be included as covariates in the subsequent regression models. After examining randomization, we will explore intervention effects. For the second aim, we will conduct linear regression to assess intervention effects on each outcome (nine models) and will covary for the baseline level of the outcome variable, intervention credibility/expectancy [23], age, sex, and race.

For the third aim, we will analyze qualitative data using the rigorous and accelerated data reduction (RADaR) technique [32]. The RADaR technique involves identifying an overarching research question, developing an all-inclusive data table from interview transcripts, and revising the table to reduce the data by retaining only the data that can answer the research question. This overall process is completed for each research question. The overarching research questions for this qualitative analysis are as follows: what do participants like about the long COVID group intervention and the participant workbook, what do they dislike about the long COVID group intervention and the participant workbook, and what are their suggestions to improve the intervention’s feasibility, acceptability, appropriateness, and efficacy? Qualitative findings will contextualize the primary outcomes data to inform specific modifications to improve the feasibility, acceptability, and appropriateness of the intervention.

We will independently and collectively review interview transcripts. Step 1 of the RADaR technique (transcription) will be completed by Rev, a HIPAA-compliant online transcription service [31]. Then a research team member will complete Step 2 of combining the individual transcripts to create an all-inclusive data table. We will complete Step 3 individually to reduce the data to a more concise data table that answers a single research question and includes notes and preliminary codes. We will then review, compare, and discuss the Step 3 data tables to select which text chunks will move forward in the analysis of the particular research question and to develop consensus on codes that will likely remain in the code column throughout subsequent steps and inform subsequent theme discussion and identification. A research team member will produce a revised data table based on the team consensus from the meeting. This process will be repeated (i.e., repeat Step 3 individually and discuss the data tables to achieve consensus) and generate and refine themes related to the research question as many times as necessary to agree the remaining data is sufficient for answering the particular research question. We will repeat the RADaR technique for each research question. We will then produce a report for each question that contains themes and exemplar quotes to help guide intervention refinement. Rigor for the RADaR technique is achieved by mapping the information in each data table phase between the individual team members and across the data table reduction phases. Thus, the iterative process involves close assessment of each analyst’s reviewing, reducing, and coding styles. Additionally, we will adhere to the standards for reporting qualitative research throughout data collection, analysis, synthesis, and interpretation and report these standards in the resultant manuscript [33]. Trial results will be disseminated via presentation at local and national conferences, peer-reviewed manuscript publications, and reporting on ClinicalTrials.gov.

### Trial status

The UW IRB approved the study protocol on October 12, 2022. The trial was posted to ClinicalTrials.gov on December 20, 2022, with the identifier NCT05658536. Recruitment started in January 2023. We expect to complete the treatment phase of the study in January 2024 and to complete assessments and qualitative interviews by February 2024.

## Discussion

This study seeks to address the urgent need for psychosocial intervention for people living with long COVID, a novel and potentially disabling medical condition. It aims to examine the feasibility, acceptability, appropriateness, and preliminary efficacy of a 6-week telemedicine group-based self-management intervention designed to improve symptom management and coping in adults with long COVID. To our knowledge, there are only a handful of RCTs examining psychosocial intervention for long COVID [34, 35]. Nevertheless, the National Institutes of Health have issued multiple funding opportunity announcements for research to improve psychosocial outcomes in people living with long COVID. This pilot RCT will advance research on psychosocial intervention for long COVID.

The study has multiple strengths. Much of the existing long COVID research examines the biological effects of the virus and focuses on medical interventions. While medical intervention research is essential, recent literature has stressed the importance of considering the psychological correlates of long COVID, particularly because psychological distress is both a symptom of long COVID and a risk factor for long COVID [17, 36, 37]. Similar to many other chronic health conditions, long COVID involves a constellation of biological/physical and psychological symptoms. Therefore, a strength of the current study is the focus on psychological correlates of long COVID, both for rehabilitation via the intervention and assessment via study outcome measures.

A second strength is our approach informed by the National Institutes of Health (NIH) Stage Model of Behavioral Intervention Development [38]. The trial focuses on Stage 1 research activities including intervention refinement and pilot testing. These activities are most appropriate as this is the first trial in a program of research aimed at developing and optimizing a self-management intervention for adults with long COVID. Although the intervention is based on evidence-based self-management treatments for other rehabilitation patient populations (e.g., multiple sclerosis) and is already being delivered at the UW due to high need and demand, an iterative multistage approach is necessary to test and refine the intervention to be maximally effective and implementable in the intended treatment setting. Additionally, the trial is pragmatic, allowing us to examine intervention feasibility and acceptability in the intended treatment setting — an overburdened long COVID clinic in an AMC setting with AMC providers. This increases the external validity of the feasibility and acceptability outcomes and subsequent intervention refinements. The NIH Stage Model of Behavioral Intervention Development is a valuable compass guiding this trial and program of research on psychosocial intervention to improve symptoms and coping in adults with long COVID.

A mixed-methods approach is another strength of this study. Mixed methodology offers greater breadth and depth to assess and contextualize study outcomes [39]; is particularly useful when examining complex and multifactorial topics, including health-focused interventions and chronic illness [40]; and helps generate and guide future research questions and ideas [41]. In the current study, qualitative data will be collected to supplement and enrich the quantitative findings on intervention feasibility, acceptability, appropriateness, and exploration of preliminary efficacy. Furthermore, the combination of quantitative and qualitative data is essential for refining and optimizing the telemedicine group-based self-management intervention, thus benefiting future long COVID clinic patients and future long COVID intervention research.

Last, the telemedicine delivery platform and group format for the self-management intervention are strengths aimed at promoting intervention reach, implementation, and maintenance in the current AMC setting as well as future adoption in other AMCs [42]. Telemedicine delivery has the potential to improve access to the intervention, particularly for patients facing barriers to in-person treatment such as geographical barriers to in-person treatment (e.g., living in rural areas), socioeconomic barriers (e.g., limited access to reliable transportation), and/or other barriers such as symptom severity (e.g., cognitive or mobility impairment), and social distancing recommendations and related fear of COVID-19 infection [13, 43, 44]. Since the COVID-19 pandemic, telemedicine has become more routine following its rapid increase due to alterations to Medicare restrictions and reimbursement of telemedicine services [44]. Additionally, the group format of the self-management intervention has been shown to promote social support and feelings of validation and to reduce isolation [45, 46]. Importantly, there is evidence that group-based video teleconference interventions produce similar outcomes to in-person group treatment [47].

Some potential limitations must be acknowledged. The study is being conducted within a single AMC, which could limit the generalizability of the findings (e.g., due to the limited patient demographics). The sample size is adequate for a mixed-methods examination of feasibility and acceptability outcomes and is appropriate for *exploration* of intervention efficacy rather than *evaluation* using fully powered statistical analysis. Last, prospective participants are identified through the wait-list for the existing telemedicine group self-management intervention. Thus, they are likely to be more amenable to the intervention and delivery format compared to patients who did not indicate interest in the group and/or declined a provider referral to the group.

## Conclusion

Long COVID poses a serious public health concern as 1 in 13 American adults report experiencing long COVID symptoms. This pilot RCT will advance rehabilitation for patients with long COVID by examining and enhancing a telemedicine group intervention designed to improve symptom management and coping in adults with long COVID.

### Supplementary Information

Additional file 1: SPIRIT 2013 Checklist: Recommended items to address in a clinical trial protocol and related documents.

Additional file 2: Supplemental Fig. 1. Sample Size Plot For Estimation of Dichotomous Feasibility, Acceptability, and Appropriateness Outcomes.

## Data Availability

Data sharing is not applicable to this protocol article as no datasets have been analyzed yet. For the study we describe, we will make the data and associated documentation available to users only under a data-sharing agreement that provides for the following: (1) a commitment to using the data only for research purposes and not to identify any individual participant, (2) a commitment to securing the data using appropriate computer technology, and (3) a commitment to destroying or returning the data after analyses are completed.
