# Supplementary material for: A pilot randomized controlled trial of a telemedicine psychosocial intervention to improve symptom management in adults with long COVID: the COPE study protocol
Source: Pilot Feasibility Stud. 2024 Jun 17;10:93. doi: 10.1186/s40814-024-01515-2 (PMC11181592; doi:10.1186/s40814-024-01515-2)

Supplemental Figure 1. Sample Size Plot For Estimation of Dichotomous Feasibility, Acceptability, and Appropriateness Outcomes


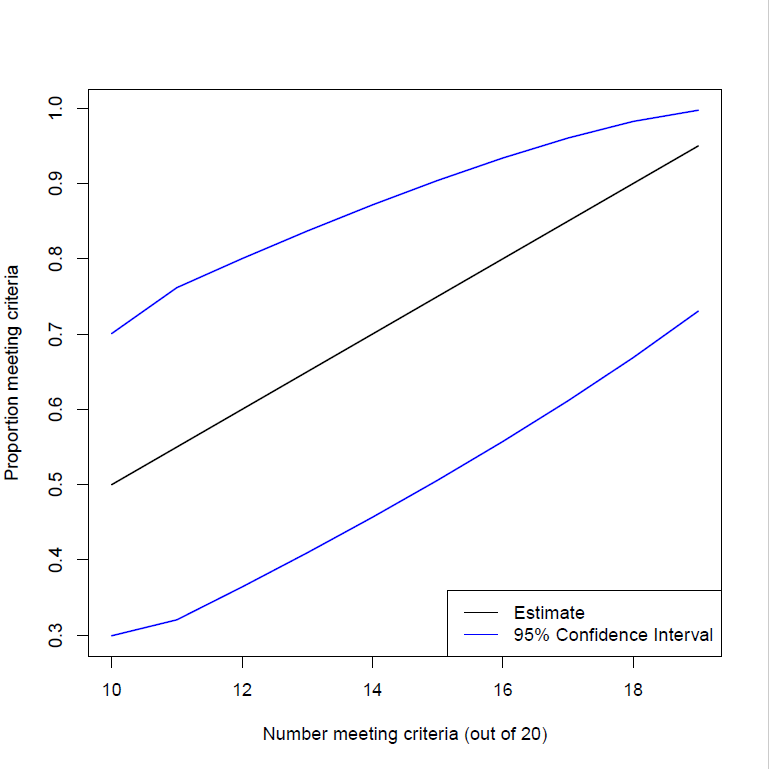

Supplement: Supplementary file 2 — Additional file 2: Supplemental Fig. 1. Sample Size Plot For Estimation of Dichotomous Feasibility, Acceptability, and Appropriateness Outcomes. [file 40814_2024_1515_MOESM2_ESM.docx]
